# The Central Roles of Noncoding RNA in Estrogen-Dependent Female Reproductive System Tumors

**DOI:** 10.1155/2021/5572063

**Published:** 2021-05-24

**Authors:** Jiajie Tu, Huan Yang, Lei Jiang, Yu Chen, Yu Chen, Zhe Li, Lei Li, Yuanyuan Zhang, Xiaochun Chen, He Chen, Zhiying Yu

**Affiliations:** ^1^Key Laboratory of Anti-Inflammatory and Immune Medicine, Ministry of Education, Anhui Collaborative Innovation Center of Anti-Inflammatory and Immune Medicine, Institute of Clinical Pharmacology, Anhui Medical University, Hefei, China; ^2^Department of Gynecology, Shenzhen Second People's Hospital, The First Affiliated Hospital of Shenzhen University Health Science Center, Shenzhen, China; ^3^Department of Obstetrics and Gynecology, The First Affiliated Hospital of USTC, Division of Life Sciences and Medicine, University of Science and Technology of China, Hefei, China; ^4^The First Clinical Medical College of Southern Medical University, Guangzhou, China

## Abstract

The pathogenesis of ovarian and endometrial cancers is closely associated with estrogen-related pathways. These estrogen-dependent tumors seriously threaten the health and quality of life in women. Noncoding RNAs (ncRNAs) are defined as RNAs that do not encode proteins, including microRNAs (miRNAs) and long noncoding RNAs (lncRNAs), both of which have been reported in estrogen-dependent female reproductive system tumors. This review systematically summarizes the role of ncRNAs in estrogen-dependent tumors and common patterns of regulatory mechanisms to explore their future research directions in tumor diagnosis, treatment, and prognosis. This may provide new ideas for the potential application of ncRNAs in estrogen-dependent female reproductive system tumors.

## 1. Introduction

Estrogen is closely related to the occurrence and development of reproductive system tumors in women, such as ovarian and endometrial cancers, which are important causes of morbidity and mortality [1]. It is well known that the occurrence of endometrial cancer is associated with long-term estrogen stimulation and estrogen receptor dysfunction [2]. Ovarian cancer is another malignant gynecological tumor with a low early diagnosis rate and high mortality rate [3]. Estrogen also plays an important role in the occurrence of ovarian cancer, but the molecular mechanism and signal transduction pathway remain elusive. Estrogen receptor (ER) is an important regulator of estrogen-dependent gynecological tumors and acts as a ligand-dependent transcription factor that participates in the occurrence and progression of tumors by regulating the transcription of specific target genes. It also indirectly modulates transcriptional regulation via the second messenger to promote cell proliferation or alleviate apoptosis of cancer cells. The activated estrogen-ER complex regulates a variety of genes that play an essential role in the cell cycle. However, the specific role of estrogen in these molecular mechanisms, such as upstream and downstream regulatory factors, is not fully understood.

Over 90% of human genomic DNA can be transcribed into RNA, of which only ∼2% is translated into proteins, with the remaining 98% being noncoding RNA (ncRNA) [4]. For the investigation of malignant tumors, most studies have focused on abnormal changes in coding genes. However, in recent years, increasing attention has been paid to the regulatory role of ncRNAs, such as microRNAs (miRNAs) and long noncoding RNAs (lncRNAs), in tumor development [5]. Most miRNAs combine with AGO2 protein to form an RNA-induced silent complex (RISC), pairing with the 3′ untranslated region of the target gene to inhibit mRNA transcription or induce its degradation. Currently, the main functions of lncRNAs include [1] participation in remodeling and modification of chromatin as a trans- or cis-regulator [2] combining with corresponding transcription factors to form a transcription complex to regulate the transcription of downstream gene targets, [3] direct involvement in the posttranscriptional regulatory process of mRNA, and [4] interaction with miRNAs as a miRNA sponge. These two main types of ncRNAs directly or indirectly participate in the regulation of oncogenes or tumor suppressors in the development of tumors and are potential targets for the prevention and treatment of cancer.

Emerging research has demonstrated that ncRNAs are abnormally expressed and play an important role in estrogen-dependent female reproductive system tumors, but most are individual studies in a particular type of tumor. Therefore, we summarize the relevant literature to review the role and compare the common regulatory pattern of ncRNAs in estrogen-dependent female reproductive system tumors. In addition, the future research direction of ncRNAs in the diagnosis and prognosis of related tumors is also discussed.

## 2. Role of ncRNAs in Estrogen-Dependent Female Reproductive System Tumors

### 2.1. Ovarian Cancer

The oncogenic effects of estrogen are mediated via both receptor-dependent and non-receptor-dependent manners [1]. In the receptor-dependent approach, estrogen binds to the nuclear estrogen receptor and causes transcription activation of the estrogen response genes, providing a signaling cascade for cell division and differentiation. These genes include several key oncogenes, such as c-fox, c-myc, and HER2/neu, as well as cell-cycle regulatory proteins and growth factors [2]. The binding between estrogen and membrane-binding G-protein-coupled estrogen receptors (GPER) activates the second messenger system. As a result, estrogen exerts a fast, nongenomic effect via GPER. The second mechanism is nonreceptor-dependent, and the formation of reactive metabolites via cytochrome P450 enzyme (CYP) may result in the generation of DNA mutation compounds. Free radicals produced by estrogen metabolism can also cause mutations, which in turn cause the accumulation of mutations in various genes in the fallopian tubes and ovarian cells. This can lead to tumor-like transformation of cells. However, all these functions of estrogen are based on coding genes. To further investigate the specific role of ncRNAs, we explored upstream and downstream regulators and other functional partners of estrogen action in female reproductive system tumors from the perspective of ncRNAs.

#### 2.1.1. The Relationship between microRNAs and Estrogen in Ovarian Cancer

Recent studies have shown that many estrogen-related tumors show dysregulated expression patterns of miRNAs [6]. MiRNAs play an important role in the development of ovarian cancer, and certain miRNAs can act as oncogenes or tumor suppressors. Dicer is an essential enzyme during miRNA processing that is required for the production of mature miRNAs. Dicer decreased in ovarian carcinomas and downregulated Dicer correlated significantly with reduced patient survival in serous cancers and advanced disease stages. In addition, reduced Dicer is associated with a global alteration of many miRNAs and genes, particularly reduced expression of ER-related genes in ovarian cancer. This suggests that Dicer-dependent biogenesis of individual miRNAs is essential for ER function in ovarian cancer [7].

Cheng et al. [8] showed that two estrogen-induced transcription factors, E2F transcription factor 6 (E2F6) and enhancer of Zeste 2 Polycomb Repressive Complex 2 Subunit (EZH2) inhibited miR-193a via an endogenous competitive RNA (ceRNA) mechanism in ovarian cancer cells. It has been further verified that the estrogen-mediated E2F6-miR-193a-EZH2 network could promote stemness of ovarian cancer cells via in vitro and in vivo experiments. Ma et al. [9] proved that estrogen promotes the expression of Olfactomedin 4 (OLFM4) by repressing miR-486-5p in ovarian cancer cells. In addition, knockdown of OLFM4 induced proliferation, invasion, and migration of ovarian cancer cells. Xie et al. [10] confirmed that the combined use of estrogen and progesterone promoted expression of let-7a and miR-34b and reduced B-cell lymphoma 2 (Bcl-2) in ovarian cancer cells, leading to inhibition of survival and promotion of apoptosis of ovarian cancer cells. When using miRNA inhibitors to suppress endogenous expression of let-7a and miR-34b, the combination of estrogen and progesterone did not change the protein level of Bcl-2 in ovarian cancer cells, suggesting that microRNA let-7a and miR-34b play key roles in the clinical treatment of ovarian cancer using estrogen and progesterone replacement therapy. Estrogen receptor alpha (ER*α*) is a direct target of miR-206 [11]. Li et al. [12] showed a significant reduction of miR-206 in ER*α*^+^ ovarian cancer tissue compared to normal ovarian epithelial tissue. Additional experiments have shown that estrogen-dependent oncogenic effects in two ovarian cancer cell lines, CAVO-3 and BG-1, can be reversed by introducing miR-206 mimics in ER*α*^+^ovarian cancer cells.

A series of studies by the Lim team found that estrogen promotes the expression of three oncogenes, Wnt family member 4 (Wnt4), avian beta-defensin 11 (AvBD-11), and secreted phosphoprotein 1 (SPP1), in hen fallopian tubes. Accordingly, three miRNAs (miR-1786, miR-1615, and miR-140) directly inhibited the expression of these three oncogenes [13–15]. Further studies showed that WNT4, AvBD-11, and SPP1 were expressed in the epithelial glands of cancerous ovaries but downregulated or were not expressed in normal hen ovarian cells.

These results suggest that multiple miRNAs repress estrogen-dependent ovarian cancer via targeting of various oncogenes, providing new potential targets and ideas for in-depth research that may lead to clinical treatments of ovarian cancer. These miRNA dysfunctions play a role in the molecular pathogenesis of estrogen-induced ovarian cancer, which may help develop new targeted therapeutic approaches for ovarian cancer.

#### 2.1.2. The Interaction between lncRNAs and Estrogen in Ovarian Cancer

Another group of ncRNAs, LncRNAs, also play an important role in gene expression and regulation in ovarian cancer through a variety of regulatory methods, such as transcription interference and chromatin modification.

A microarray-based high-throughput study was performed to identify estrogen-regulated lncRNAs in ER*α*^+^ ovarian cancer cells. One hundred and fifteen lncRNAs exhibited significant changes in the estrogen-treated ovarian cancer line, SKOV3. Three lncRNAs (TC0100223, TC0101686, and TC0101441) demonstrated correlations with typical malignant cancer phenotypes, such as advanced FIGO stage and/or high histological grade. In addition, TC0101441 was shown to be an independent prognostic factor for overall survival. These results indicate that estrogen can modulate lncRNA expression in ER*α*^+^ ovarian cancer cells, and that certain lncRNAs are correlated with advanced cancer progression and are a suggestive prognostic indicator in ER*α*^+^ ovarian cancer patients. Knowledge of these estrogen-regulated lncRNAs could help our understanding of the estrogenic effect on ovarian cancer and may assist in the clinical design of new target therapies based on lncRNA [15]. In light of the above high-throughput study, Ye et al. [16] confirmed that lncRNA TC0101441 has a promotive effect on the migration of endometrial cancer cells in vitro. Knockdown of TC0101441 partially damaged estrogen-induced migration and invasion of endometrial cancer by regulating the matrix metalloproteinases MMP-2 and MMP-3. It has been shown that estrogen also induces TC0101441 via ER*α*-estrogen reaction element (ER*α*-ERE) binding [17]. An in vitro knockdown experiment found that ElncRNA1 promoted the proliferation of endometrial cancer cells by regulating cell cycle-dependent kinases (CDK4 and CDK6) and G1/S-specific periodic protein-D1 (cyclin D1).

Depletion of lncRNA LINC00511 enhanced cell growth and invasion and reduced the apoptosis rate of CAOV3 cells [15]. 17 beta-estradiol stimulation of ESR1 (ER*α* gene) increased the expression of lncRNA LINC00511, while the ESR1 inhibitor fulvestrant reduced expression of lncRNA LINC00511 in CAOV3 cells. It was predicted that lncRNA LINC00511 interacts with miR-424-5p and miR-370-5p via bioinformatics. These results suggest that ESR1-induced upregulation of lncRNA LIN00511 may promote proliferation and invasion of CAOV3 cells by regulating miR-424-5p and miR-370-5p.

**Figure 1:**
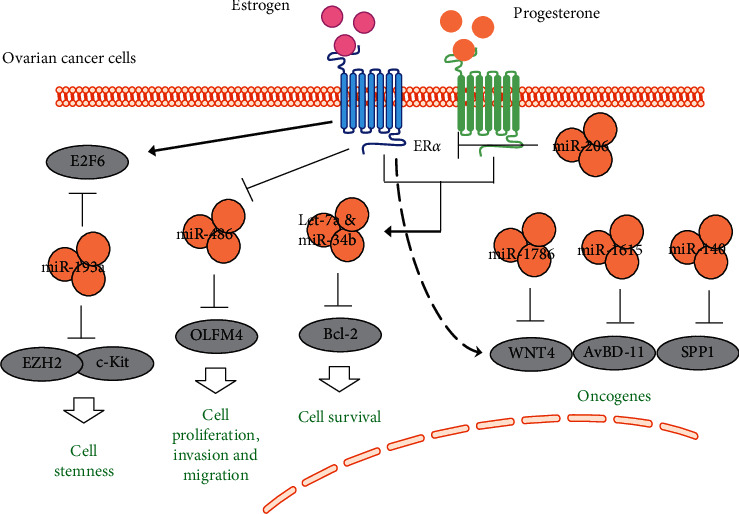
The interaction of miRNAs and estrogen in ovarian cancer. Several miRNAs have been reported that interact with estrogen and its receptor in ovarian cancer, including miR-193a, miR-486, let-7a, miR-34, and miR-206. Solid arrow: induction; flat-ended arrow: inhibition.

**Figure 2:**
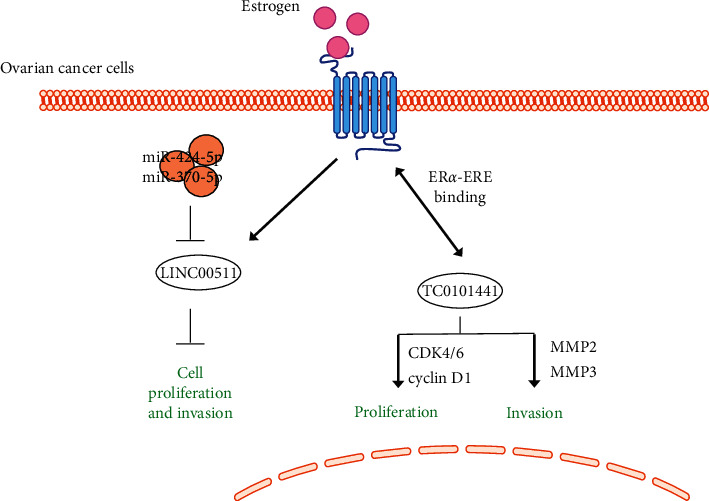
The interaction of lncRNAs and estrogen in ovarian cancer. Two lncRNAs, LINC00511 and TC0101441, have been reported to interact with estrogen and its receptor in ovarian cancer. Solid arrow: induction; flat-ended arrow: inhibition.

Understanding the molecular basis of ER*α* expression is critical to develop novel targets to inhibit ovarian cancer. In this section, we summarize ER*α*-related miRNA and lncRNA in ovarian cancer. Many ncRNA may be effective inhibitors of ER*α* function, such as miR-206, which directly inhibits the expression of ER*α*in ovarian cancer cell line. In addition, the mechanism of estrogen-ncRNA interaction in ovarian cancer is not through a single pathway, such as DEC-induced WNT4 and AvBD-11 via miR-1786 and miR-1615 to induce tumor occurrence, respectively. On the contrary, several ncRNAs can also promote tumor progression via the same targets. For example, both estrogen and progesterone-induced let-7a and miR-34b can promote apoptosis and repress survival of tumor cells by inhibiting Bcl-2 (Figure 1). The same is true for lncRNAs. As shown in Figure 2, estrogen can raise TCO101441 to promote tumor development via ER*α*-ERE binding. This single lncRNA can exert its bifunctional effects via two pathways: CDK4, CDK6, and cyclin D1 for cell proliferation, and MMP-2 and MMP-3 for cell invasion and migration. In summary, ncRNAs may be involved in estrogen-mediated progression of endometrial cancer through multiple mechanisms, which will provide us with more ideas and directions for clinical treatment and drug development. Since inhibition of ER function is a key therapeutic option in estrogen-dependent ovarian cancer, these results may provide new insights into mechanisms to inhibit progression of ovarian cancer.

### 2.2. Endometrial Cancer

A clear understanding of the specific role of estrogen is essential for the pathogenesis of endometrial cancer. Previous studies have found that the accumulation of DNA replication errors during mitosis can lead to malignant transformation in actively proliferating cells. Both normal endometrial glands and epithelial cells express estrogen receptors and can proliferate upon estrogen stimulation. Therefore, long-term exposure to estrogen plays a key role in the cancerization of endometrial epithelial cells.

Estrogen mainly exerts its oncogenic effects in endometrial epithelial cells from two aspects: [1] the lack of DNA repair systems in actively replicating cells and [2] estrogen-derived metabolites that may cause mutations. Therefore, high levels of estrogen are thought to stimulate the development of endometrial cancer. However, some clinical data suggest that most endometrial cancer occurs at the perimenopause stage when estrogen levels decline in serum, which is inconsistent with existing results [16]. Thus, the actual effect of estrogen on endometrial cancer has not yet been fully clarified, suggesting that we should explore the molecular mechanisms of estrogen in endometrial cancer from a new perspective of ncRNA.

#### 2.2.1. The Relationship between miRNAs and Estrogen in Endometrial Cancer

Endometrial cancer is one of the most common malignant tumors in the female reproductive system, and estrogen plays an important role in the pathogenesis of endometrial cancer.

Tamoxifen is a selective estrogen receptor modulator that has been widely used in the treatment of hormone-responsive breast cancer [18]. The estrogen-like effect of tamoxifen increases the risk of endometrial cancer [19]. When treated with tamoxifen, invasiveness and epithelial-mesenchymal transition (EMT) were induced in endometrial cancer cells. MiR-200 was found to be reduced in response to tamoxifen treatment. A number of key factors of EMT, such as zinc finger E-box binding homeobox 2 (EZH2), Snail, N-cadherin, and E-cadherin, were modulated by miR-200 in tamoxifen-treated endometrial cancer cells. EZH2 was also verified as a direct target of miR-200 in endometrial cancer cells. In addition, c-Myc has been proven to be a transcriptional inhibitor of miR-200 [20]. Another study showed that PTEN (tumor suppressor) and PTENP1 (PTEN homologous) are two direct targets of miR-200 in endometrial cancer cells [18]. Therefore, estrogen also regulates miR-200-PTEN/PTENP1 by binding ER*α* and then modulates endometrial cancer viability and aggressiveness.

PTEN is regulated by miR-200 in endometrial cancer. MiR-181c also induces cell viability and represses the apoptosis in the estrogen-induced endometrial cancer cell line RL95-2. Overexpression of PTEN reversed the inhibitory effect of miR-181c on apoptosis by regulating Bax and Bcl-2 expression and modulated the activity of the AKT pathway, p53, and cyclin D. PTEN was also shown to be a direct target of miR-181c in RL95-2 cells [21].

It is known that estrogen can induce an imbalance in BCL2/BAX expression in endometrial cells, leading to precancerous lesions and type I endometrial adenocarcinoma. Activated ER suppresses BAX by upregulating several miRNAs, including let-7 family members and miR-27a, thereby promoting an increased BCL2/BAX ratio as well as enhanced survival and proliferation in the affected cells. ER-induced let-7 could be detected in most hyperplastic endometria, suggesting their potential utility as indicators of uncontrolled estrogen exposure [22].

miR-195 inhibited the viability and migration of two endometrial cancer cell lines, AN3-CA and Hec1A cells. The phosphorylation levels of PI3K and AKT were also repressed. G-protein-coupled estrogen receptor 1 (GPER) was identified as a direct target of miR‐195. The inhibitory effects of miR-195 on endometrial cancer cell migration and invasion are via the PI3K/AKT signaling pathway and GPER [23].

Estrogen induced miR-203 in the rat endometrial adenocarcinoma cell line RUCA-I. Proliferation was reduced and G2-arrest was observed in miR-203 knockout RUCA-I cells. RNA-seq demonstrated that miR-203 upregulated 566 genes and downregulated 592 genes in RUCA-a cells, respectively. Many of the significantly changed genes were closely related to the estrogen signaling pathway. Interestingly, of these estrogen-related genes, *Acer2*, *Zbtb20*, *Ptn*, *Rcbtb2*, *Mum1l1*, *Hmgn3*, and *Nfat5* possessed one or more seed sequences in their 3′-UTR that were predicted to be targets of miR-203. These data demonstrate that estrogen exerts its oncogenic effects in the etiology of endometrial carcinomas by inducing miR-203 [24].

miR-30c was downregulated in endometrial cancer tissue and highly expressed in the ER-negative endometrial cancer cell line HEC-1-B. MiR-30c directly inhibited metastasis-associated gene-1 (MTA-1) expression and functioned as a tumor suppressor via the miR-30c-MTA-1 signaling pathway. Furthermore, miR-30c treatment decreased upon estrogen expression in endometrial cancer cells, suggesting that estrogen-induced miR-30c is an important deregulated miRNA in endometrial cancer [25].

Studies have shown that miR-152 regulates the cell cycle [26]. Nie et al. [19] showed that progesterone (P4) induces the expression of miR-152 in ovary-removed mice and the endometrial cancer cell line Ishikawa. The expression of miR-152 was upregulated in P4 receptors overexpressing human endometrial cancer cells. By using miRNA mimics and inhibitors, it was proved that miR-152 can block G1/S conversion in endometrial epithelial cells (EEC) and inhibit cell proliferation by targeting WNT-1 in endometrial cancer cells, suggesting that miR-152 is a potential anticancer miRNA in endometrial cancer.

**Figure 3:**
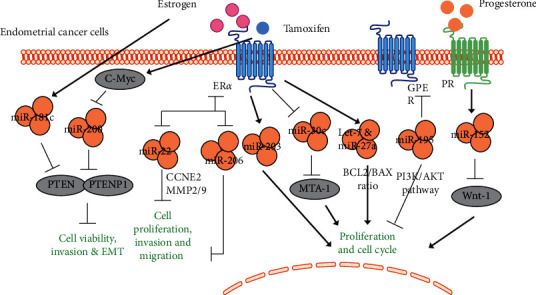
The interaction of miRNAs and estrogen in endometrial cancer. Several miRNAs have been reported to interact with estrogen and its receptor in endometrial cancer, including miR-181c, miR-200, miR-22, miR-206, miR-203, miR-30c, Let-7, miR-27a, miR-195, and miR-152. Solid arrow: induction; flat-ended arrow: inhibition.

Li et al. [20] found that miR-22 was downregulated in ER*α*-positive EEC tissues and cell lines compared to normal endometrial and ER*α*-negative EEC. MiR-22 overexpression inhibited ER*α* expression in RL95-2 human endometrial cancer cells and Ishikawa cells. This study further proved that the inhibitory effects of miR-22 on proliferation, migration, and invasion of these ER*α*-positive EEC lines were at least partially mediated by inhibiting the expression of cyclin E1 (CCNE1) and the secretion of matrix metalloproteinases MMP-2 and MMP-9. These results suggest that miR-22 is a potential candidate for ER*α*-positive EEC therapy. The expression of miR-206 was repressed in 30 clinical samples of EEC [21]. Results of the luciferase reporter assay showed that ER*α* is a direct target of miR-206. Further studies found that miR-206 expression in ER*α*-positive EEC was negatively correlated with ER*α*, and miR-206 overexpression inhibited ER*α*-dependent proliferation, invasion, and induced cell cycle blockage. Understanding these estrogen-related miRNAs provides new awareness and potential therapeutic targets for EEC treatment from the perspective of miRNAs (Figure 3).

#### 2.2.2. The Interaction between lncRNAs and Estrogen in Endometrial Cancer

Studies have shown that estrogen regulates lncRNAs in ER*α*^+^ endometrial cancer. Some lncRNAs are associated with advanced cancer progression and can indicate the prognosis of patients with ER*α*^+^ endometrial cancer. Therefore, understanding these estrogen-regulated lncRNAs can help us to understand the effects of estrogen on the progression of endometrial cancer and may provide new targets for the clinical treatment of endometrial cancer.

HOTAIR is a potential predictor of poor prognosis in four of the main estrogen-dependent tumors, especially in cervical, ovarian, and endometrial cancer patients without preoperative treatment in Asian populations [27]. Specifically, HOTAIR expression was negatively related to miR-646 in human endometrial cancer tissues. HOTAIR promoted the viability, migration, and invasion of endometrial cancer cells by negatively regulating miR-646. In addition, nucleophosmin 1 (NPM1) was shown to be a target of miR-646. Therefore, the HOTAIR-miR-646-NPM1 ceRNA regulatory axis is involved in the progression of endometrial cancer [28].

The expression of ncRNA NIFK-AS1 decreased and miR-146a increased in primary tumor-associated macrophages of endometrial cancer patients. NIFK-AS1 overexpression reversed IL-4-induced M2 polarization of THP-1 macrophages and indirectly inhibited estrogen-induced proliferation, migration, and invasion of endometrial cancer cells in a coculture system *in vitro*. NIFK-AS1 interacts with miR-146a and increases the expression of Notch1 by downregulating miR-146a. miR-146a overexpression attenuated the effect of NIFK-AS1 on suppressing M2 polarization of macrophages and estrogen-induced proliferation, migration, and invasion of endometrial cancer cells. This study provides novel insights about NIFK-AS1 in the regulation of polarization and function of tumor-associated macrophages in endometrial cancer [29].

**Figure 4:**
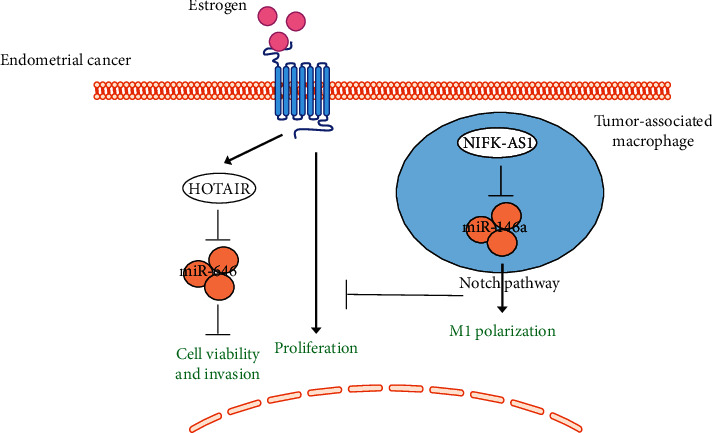
The interaction of lncRNAs and estrogen in endometrial cancer. Two lncRNAs, HOTAIR and NIFK-AS1, have been reported to interact with estrogen and its receptor in endometrial cancer via miRNA sponging. Solid arrow: induction; flat-ended arrow: inhibition.

Current studies have demonstrated that ncRNAs play a multifunctional role in EC (Figure 4). However, there are still many questions that need to be addressed before future application. Can ncRNAs be used as a biomarker for EC diagnosis and as an indicator of EC metastasis? Does modulation of individual ncRNA and its associated pathways help to cure or control EC progression? Both miRNAs and lncRNAs show specific expression patterns in EC and are associated with some known carcinogenesis factor of EC, such that estrogen could directly induce miR-181c and HOTAIR in EC. Therefore, the effects of ncRNAs on estrogen-induced EEC-EC transition should be systematically clarified in the future. Although the study of ncRNAs in EC is still at relatively early stage, to investigate its role in EC will further deepen our understanding of EC's occurrence, development, diagnosis, and treatment.

### 2.3. Hysteromyoma

The uterus is an essential organ of the female reproductive system and is the place of fetal growth. Uterine fibroids are common benign tumors in women. The main clinical manifestations include uterine bleeding and abdominal swelling, seriously affecting the health of women. It is well known that uterine fibroids are estrogen-dependent tumors, and estrogen is involved in the development of uterine fibroids by binding to the ER. Except for hysterectomy, most current treatments for uterine fibroids are usually temporary and not successful for all patients. Therefore, the specific role of estrogen in uterine fibroids requires further investigation. Deng et al. [23] have shown that ovarian estrogen plays a key role in the pathogenesis of smooth fibroids by regulating miRNAs.

A number of studies [30, 31] have shown that miR-129 is downregulated in a variety of tumors and is involved in the regulation of tumor development. By using the dual-luciferase reporter and western blot assays, the regulatory relationship between miR-129 and the target gene TET1 (one of the members of the TET protein family) was verified. Transfection of miR-129 mimics and TET1 siRNA inhibited cell proliferation and migration and promoted apoptosis of uterine fibroid cells. MiR-129 expression was repressed by estrogen and progesterone, and its downregulation was beneficial to the development of uterine fibroids. TET1 is known to be an important enzyme in DNA demethylation, which is a critical epigenetic modification [32]. These studies suggest that further study of miR-129-TET1 and DNA demethylation in the apoptosis pathway will provide novel ideas for exploring the mechanism and treatment of uterine fibroids.

The miR-29 family consists of miR-29a, miR-29b, and miR-29c, which have a common seed sequence, but each has a unique functional activity [28]. Dyrskjøt et al. [30] showed that miR-29c expression was inhibited in uterine fibroids and its expression was negatively correlated with the expression of its target genes, CL3A1 and DNMT3A. The inhibition of miR-29c in smooth fibroids was mediated by epigenetic mechanisms and transcriptional regulation of NF-*κ*B and SP1. MiR-29c and its target genes regulate a variety of cellular activities, such as cell proliferation and angiogenesis, which are at the core of the development of uterine fibroids. In addition, studies have shown that the expression of miR-29c is regulated by estrogen and progesterone. These results suggest that the NF-*κ*B/SP1-miR-29c- CL3A1/DNMT3A axis is essential in steroid-mediated uterine fibroids.

HPV16 E7 oncoprotein in conjunction with estrogen is sufficient to produce high-grade cervical dysplasia and invasive cervical malignancies in a mouse model. MiR-21 was upregulated and miR-143 was downregulated by the HPV16 E7 oncoprotein in vivo and in vitro. Estrogen treatment is also implicated in the deregulation of these important miRNAs in vivo. PTEN and Bcl-2 were identified as two direct targets of miR-21 and miR-143, respectively. These results suggest that HPV type 16 E7 oncoprotein and estrogen play an important role in regulating miR-21 and miR-143 expression [33].

LncRNA SRA1 is known to enhance the transcriptional activity of estrogen receptors and promote steroidogenesis. Mutations were detected in exon 2 of MED12 in 28 uterine leiomyoma samples (75% missense mutations and 25% in-frame deletions). Expression of SRA1 was higher in uterine leiomyoma samples without MED12 mutations than in uterine leiomyoma samples harboring MED12 mutations. The present results suggest that SRA1 may explain the phenotypic difference observed in the tumor sizes of uterine leiomyoma samples considering the MED12 mutation pattern [34].

**Figure 5:**
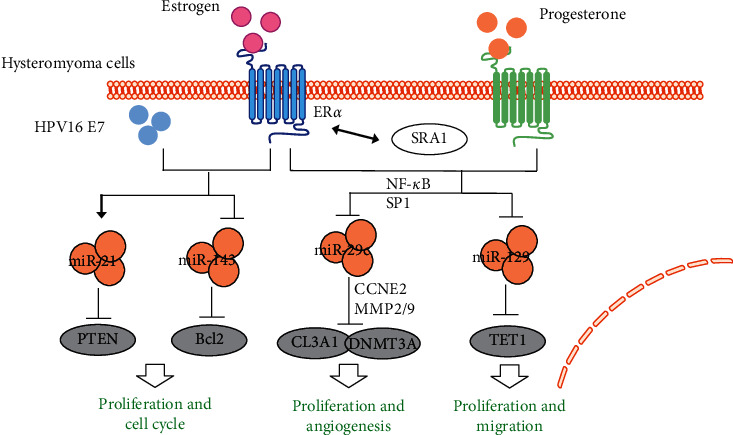
The interaction of ncRNAs and estrogen in hysteromyoma. Several ncRNAs have been reported to interact with estrogen and its receptor in hysteromyoma, including four miRNAs (miR-21, miR-143, miR-29c, and miR129) and a lncRNA SRA1. Solid arrow: induction; flat-ended arrow: inhibition.

Hysteromyoma is hormone-dependent tumor, and estrogen promotes the occurrence and development of uterine fibroids [35]. A series of articles have shown that estrogen affects many aspects of hysteromyoma, including proliferation, metastasis and angiogenesis, via regulating multiple ncRNAs. Interestingly, it has been documented that estrogen can modulate the expression of two DNA methylation-related epigenetic regulatory proteins, DNMT3A and TET1, by inhibiting miR-29c and miR-129, respectively. Therefore, the role of estrogen and DNA methylation/demethylation in the development of uterine fibroids should be studied in uterine fibroids simultaneously, and the application of 5mC-sequencing and 5hmC-sequencing can provide new ideas for the pathogenesis of uterine fibroids at the genome-wide level. In addition, since ER*α* has been shown to be an oncogenic factor in uterine fibroids, the specific mechanisms of lncRNA SRA1 and ER*α* should be further clarified. The combination of epigenetic modifications (including miRNA, lncRNA, and DNA methylation/demethylation) can provide a novel direction for understanding of pathogenesis of estrogen-dependent hysteromyoma (Figure 5).

## 3. Discussion and Perspectives

The development of many tumors that threaten women's health, such as ovarian and endometrial cancers, is closely related to estrogen levels and the activity of their receptors. Serum estrogen fluctuates dramatically throughout women's lives, and studies have shown that estrogen in women of normal reproductive age during the menstrual cycle ranges from 50 to 300 pg/mL. High estrogen levels (more than 200 pg/mL) are required to induce luteinizing hormone peaks and ovulation. During the perimenopausal and early postmenopausal periods, estrogen levels drop below 80 pg/mL, and even below 10 pg/mL in the late menopausal period. In order to be pregnant, estrogen levels in young women must rise to induce ovulation. However, if the elevated estrogen is not restricted, the risk of cancer in women may increase accordingly. As a result, young women are protected by two blocking systems in the body: one is the activation of estrogen-induced DNA mismatch repair system (MMR), and the other is estrogen-induced ovulation and subsequent progesterone secretion. Thereby, young women are protected from the potential risk of cancer caused by estrogen. In women who are perimenopausal or postmenopausal, this dual blocking system does not work because low levels of estrogen in the body do not induce ovulation conditions. Although most postmenopausal women showed relatively low estrogen concentrations, the average serum level of estrogen in cancer patients was still higher than that in normal control women. Estrogen is an important risk factor in these women. Therefore, the use of drugs or factors that increase the level of estrogen in the body should be avoided.

Deregulation of ncRNAs is one of the characteristic features observed in estrogen-dependent female reproductive system tumors and their role in regulation of the hallmarks of cancer [36, 37] mainly is associated with proliferation and metastasis. The effects of ncRNAs on other hallmarks of cancer, such as angiogenesis, genome instability, reprogramming of energy metabolism, and evading immune destruction, have been proven in other types of cancer [38–41]. In the future, the relationship between ncRNAs and these hallmarks of cancer should be investigated in estrogen-dependent female reproductive system tumors.

Estrogen-induced abnormal expression of ncRNAs can lead to corresponding changes in downstream genes, which affect tumor progression. There are many mechanisms for ncRNAs to regulate cancer cells, mainly through epigenetic mechanisms to change proteins that are essential for tumor development. This suggests that monitoring changes in ncRNA-regulated proteins in patients with estrogen-dependent tumors is a potential method for future clinical treatment and diagnosis. From the earliest discovery of the first miRNA in *Caenorhabditis elegans*, scientists determined that ncRNAs are widely involved in almost all aspects of life, such as growth, differentiation, development, immunity, and the occurrence and development of tumors [42, 43]. ncRNA research has revealed the mechanism of action of cellular activities from a new perspective and has become a hot topic in life science. Although we have made progress in understanding that ncRNAs and ncRNAs are promising potential therapeutic target of estrogen-dependent female reproductive system tumors, there are still some challenges that need to be addressed before the clinical applications, for example, the lack of *in vivo* models and the homogenization of related studies, which limit our ability to investigate the diversity of ncRNA mechanisms. In addition, “off-target effects” of miRNAs also limit their potential clinical application, which should be evaluated in future studies. Moreover, the safety of ncRNA as a therapeutic target needs to be determined. Inhibitors or activators of ncRNA are usually composed of double-stranded RNA and delivered by a virus-based system, which can lead to an overactive innate immune response. These issues need to be systematically assessed in preclinical studies. The usage of miRNA and lncRNA in the diagnosis and treatment of female reproductive tumors, such as directly targeting oncogenic miRNA or lncRNA or in combination with other existing drugs such as selective ER modulator, should be investigated in future studies.

From the studies of ncRNAs in estrogen-dependent tumors, gene regulation by ncRNAs is through a synergistic network. A variety of ncRNAs participate in the regulation of tumor development, suggesting that we should not only investigate the mechanism of action of individual ncRNAs, but also explore common targets of different ncRNAs. For example, an estrogen-ncRNA-target database can be established to discover commonalities of a certain class of ncRNA, so as to find the most effective targets for the treatment of tumors. In the treatment of estrogen-dependent tumor patients in the future, ncRNAs-target therapy is another promising option. By repressing the proliferation and migration of tumor cells from the root via targeting ncRNAs, it is believed that ncRNAs will play an important role in the treatment and prognosis of estrogen-dependent tumors in the near future, bringing new hope to cancer patients.
